# Editorial Department

**Published:** 1856-05

**Authors:** 


The Class of the New York Medical College have recently presented to Dr. Isodore Glück, a handsome case of post-mortem instruments. The following letter in reply, explains sufficiently the motives of the presentation. We give it, partly out of a desire to compliment Dr. Glück, whose talents we value very highly, but quite as much for the valuable practical suggestions it contains:

691 Broadway, March 1, 1856.

Gentlemen: Allow me to thank you for the high appreciation of the slight services rendered by me to you, expressed not only in flattering words, but accompanied by a substantial memento that, by the reminiscence of its source, will always remain very valuable to me as the greatest proof of your satisfaction. I am proud of having received it from a class, many of which are but entering practice, and have already felt the necessity of becoming familiar with studies, practical in their nature, this want of which knowledge is generally only experienced by those, who, when in practice have to apply either the one, military surgery, or the other, that of conducting post-mortem examinations, in a legal manner. The former, when called upon by railway or other accidents, often placing the physician in a more difficult position than the military surgeon, the latter equally perplexing when he is called upon to conduct post-mortem examinations, according to the requirements of this law. I am the more sensible to your regards because I was fearful that I might have been misunderstood. On comparing the systems and general arrangements for conducting post-mortem examinations in this country with those in other countries in a medico-legal point of view, I felt myself compelled to denounce them to you, gentlemen, as careless, inaccurate and insufficient—as often hindering the ends of justice and putting the public to great unnecessary expense. I have, in my lectures to you, gentlemen, endeavored to impress on you the necessity of a more accurate and severe study of medical jurisprudence, as absolutely necessary to the accomplished physician, in order to render him not only a more competent guardian of the public health, but also a valuable aid to justice, in meting out rewards to the innocent as well as the guilty. The want of dead houses expressly devoted to conducting legal post-mortem examinations, along with a regularly appointed staff of competent medical men, detailed for that especial duty, must be apparent to you. Dead houses, where men of scientific attainments and known professional skill will be compelled by law to furnish full and accurate reports, after a prescribed legal form, of all cases brought before them, whether from violence, poison, drowning, or freezing; and to whom the public will look for a strict and analytical examination of the cause of deaths in all cases brought under their inspection. When I contrasted the manner in which post-mortem examinations were conducted in this city, the scanty and insufficient accommodations allowed to the medical officer of the law for conducting such examinations, and the meagre and unsatisfactory report which is at present required at his hands, I did not do it from a captious spirit or a wish to underrate, but for the sake of bringing to your attention, and through you to the public, the manifold defects of the present system of conducting post-mortem examinations—defects which I trust you will be unceasing in your efforts to obviate or remove. It reflects great credit on the members constituting the class of the New York Medical College, that they have voluntarily abridged the short time allowed for necessary recreations, to devote themselves to the acquisition of knowledge in this most important department of their profession. It tends to prove that the rising medical generation feels the time usually prescribed for medical studies as too short and inadequate to the exigencies of the profession; that some reform in this respect is necessary, and is immediately demanded by all those who desire to see the profession of medicine honored as it deserves. It is not the multitudes of colleges, with an uniform educational programme, but the liberal tendency of a few well organized institutions, that can give the guarantees of that liberal medical education you are striving for, and which the community at large has reason to expect and a right to demand from those to whom it entrusts its public health or individual safety.

Accept, gentlemen, my most sincere wishes for your welfare, and that of your esteemed colleagues, and believe me,

Yours respectfully,

ISODORE GLUCK.

Dr. W. C. Williams and others of the committee.

